# Effect of upper eyelid blepharoplasty on corneal biomechanical, topographic and tomographic parameters 4 weeks after surgery

**DOI:** 10.1007/s10792-021-02006-6

**Published:** 2021-09-03

**Authors:** Falk Sommer, Elisa Untch, Eberhard Spoerl, Robert Herber, Lutz E. Pillunat, Naim Terai

**Affiliations:** Department of Ophthalmology, University Hospital Carl Gustav Carus, TU Dresden, Fetscherstr. 74, 01307 Dresden, Germany

**Keywords:** Upper eyelid blepharoplasty, Corneal hysteresis, Corneal resistance factor, Central and peripheral cornea, Topography, Tomography

## Abstract

**Purpose:**

To investigate the effect of “skin-only” upper eyelid blepharoplasty on corneal biomechanics and central as well as peripheral topographic/tomographic parameters before and 4 weeks after surgery.

**Methods:**

In a prospective study, the corneal hysteresis (CH) and corneal resistance factor (CRF) were evaluated before and after blepharoplasty. Corneal topographic (maximum simulated keratometry value, inferior-superior value, index of surface variance, index of vertical asymmetry, index of height asymmetry, index of height decentration) and tomographic parameters (corneal thickness, corneal astigmatism and mean 5-mm- and 7-mm-zone keratometry value) were measured by the Pentacam HR. Statistical analysis was performed using a linear mixed model considering correlated data of both eyes.

**Results:**

This study included 42 eyes of 35 patients (mean age: 64.5 years, range 52–82 years). Four weeks after surgery CH and CRF increased (9.4 ± 2.3 to 10.2 ± 2.2 mmHg and 9.7 ± 2.1 to 10.5 ± 2.2 mmHg) but did not reach statistical significance (*P* = 0.100 and *P* = 0.072). A significant increase in central maximum simulated keratometry value (Kmax) from 45.0 ± 2.3 to 45.4 ± 2.2 diopters (D) was observed (*P* = 0.004). Inferior-superior value (I-S) and index of surface variance (ISV) showed significant changes from 0.32 ± 0.98 to 0.10 ± 0.98 D (*P* = 0.02) and from 19.98 ± 9.84 to 22.93 ± 11.23 (*P* = 0.009), respectively. These alterations did not affect the subjective spherical equivalent (-0.09 ± 4.71 to -0.04 ± 4.51 D; *P* = 0.437) and the best-corrected distance visual acuity of patients (0.11 ± 0.14 to 0.15 ± 0.15 logMAR; *P* = 0.142). Age, gender and corneal thickness were not correlated with pre and postoperative differences of CH, CRF, corneal compensated IOP, Kmax, corneal astigmatism or I-S.

**Conclusion:**

The trend of increasing CH and CRF values might indicate a rise of corneal damping capacity. Despite statistically significant differences of Kmax, I-S and ISV, all other tomographical and topographical parameters did not change 4 weeks after surgery. The corneal steepening with a mean change of 0.4 diopters and the decrease of I-S with a mean of 0.22 diopters do not seem to have a clinically relevant effect for blepharoplasty patients in daily practice.

## Introduction

Worldwide blepharoplasties represent one of the most frequent surgical procedures of the eyelid. In the U.S, approximately 150.000 blepharoplasties of the upper and lower eyelid are performed annually. Aims of the upper eyelid blepharoplasty are: excision of excessive skin/fat-tissue, correction of a mild ptosis or a fat-tissue redistribution e.g., in patients with deep upper lid sulcus after long term topical prostaglandin treatment [1].

Frequently patients after surgery complain about visual changes which can also be ascribed to postoperative changes of corneal topography [2].

For lower eyelid surgery, some facts are established: changes in astigmatism and refractive power after tarsal strip procedure [3] and a shift from ‘with-the-rule’ to ‘against-the-rule’ axis, high order aberration changes [4] and astigmatism reduction due to corneal flattening in the inferior quadrant [5].

Previous studies reported on small but persistent dioptric changes (< 1 diopters) in the central and peripheral cornea in about 90% of all patients after upper eyelid blepharoplasty [6]. Eyelid surgery may alter the pressure on the cornea and hence the corneal shape and curvature [7]. The Ocular Response Analyzer (ORA) provides two parameters, corneal hysteresis (CH) and corneal resistance factor (CRF), which are related to the corneal shape and thickness [8].

Aims of the present study were: (1) To investigate whether changes of topo and tomographic parameters are correlated to changes of corneal biomechanics 4 weeks after upper eyelid “skin-only” blepharoplasty, (2) To examine surgery induced changes in topographic and tomographic corneal parameters in the early postoperative period and (3) To differentiate between peripheral and central changes of the cornea 4 weeks after upper eyelid “skin-only” blepharoplasty.

To the best of (our) knowledge, the present study is the first investigation of corneal biomechanical changes after “skin-only” blepharoplasty using the ORA, taking into account corneal surface indices.

## Methods

This prospective study was conducted at the Department of Ophthalmology of the University Hospital Carl Gustav Carus, Technical University Dresden, Germany. Institutional review board approval was obtained for all aspects of this study (ID: EK 113032017). Informed consent from the patients to use data for analysis and publication was collected and signed before participating in this study. All study related procedures adhered to the tenets outlined in the Declaration of HELSINKI. Inclusion criteria for this study was an upper eyelid “skin-only” dermatochalasis with superior visual field impairment based on following parameters in sitting position:

- Height of the palpebral aperture at the midpupil point: 8–11 mm
- Levator function > 12 mm
- Distance between upper lid margin and central pupil light reflex (margin reflex distance 1): 2.5–5 mm
- Distance from midpupil to the top of the brow: > 22 mm

Furthermore, in all patients lid closure and bell phenomenon were intact. Only patients with first blepharoplasty were included.

Exclusion criteria were a MRD 1 < 2.5 mm and > 5 mm, the presence of prolapsed upper lid fat compartments and the necessity of fat removal or resection of the orbicularis muscle, keratoconus or other corneal diseases, glaucoma and dry eye syndrome. Also, patients with muscle and nerve lesions which could have affected lid function and position were excluded.

All blepharoplasties were undertaken in infiltrative anesthesia and by a single surgeon (F.S.). After marking of excessive lid skin in the sitting and supine position with open and closed eyes, infiltration anesthesia (xylocitin® 2% with epinephrin 0.001% (1: 100 000), mibe GmbH Arzneimittel, Germany) was performed. The inferior incision with the scalpel was performed 1 mm under the natural upper lid skin crease (women: 9–10 mm, men: 8–9 mm distance from the upper lid margin), and the excision of the skin was carried out with a straight, sharp scissor. In all patients, at least 10 mm of skin was left between the lower margin of the brow and the upper incision. The length of the horizontal skin incision in all patients ranged from 25 to 35 mm, the amount of vertically excised skin was 12–16 mm at the point of the greatest width (measured while tightening the skin in supine position before skin excision). In all patients, the incision was carried out medially not exceeding the lacrimal punctum and was extended temporally beyond the orbital rim. After bipolar cauterization and wound disinfection, wound closure was performed with a continuous cutaneous suture (non-resorbable, monofile, synthetic-Prolene® 6–0, P 1 needle, Ethicon). Wound disinfection was repeated and leukostrips (Smith & Nephew, 4.0 mm × 38 mm) were applied onto the suture. Sutures were removed between the 9th and 12th day after surgery.

Biomechanical, topographic and tomographic measurements were performed before (at the day of) and 4 weeks after surgery according to standard protocols by one experienced operator. The ORA (software version 3.01, Reichert Ophthalmic Instrument, Buffalo, NY, USA) was used for biomechanical assessment. Patients were instructed to sit on a chair in front of the device. Upon successful fixation of the patient’s eye on a red blinking target, the operator activated the device. An air puff was released by a non-contact probe which scanned the central area of the eye and sent a signal to the ORA. In brief, the air puff causes the cornea to move inwards. The first applanation is followed by a phase of highest concavity. Subsequently, a second applanation occurs before the cornea reaches its initial shape again. The system monitors the entire process and produces a specific waveform [9]. Four variables are provided by the ORA: the Goldmann-correlated IOP (IOPg), the corneal compensated IOP (IOPcc), the CRF and the CH. The IOPg is calculated as the average of first (P1) and second applanation (P2) of the cornea induced by the air puff. However, the cornea shows a viscoelastic behavior as a response to the air puff applied which leads to a reduced second applanation. These differences are described as CH. CH is the result of the viscous damping within the corneal tissue which is created by the viscosity of glycosaminoglycans and proteoglycans. CRF appears to be an indicator of the overall “resistance” of the cornea [10].

Topographical and tomographical data of the cornea were investigated by the Pentacam® HR (software version 1.21r65, Oculus Optikgeraete GmbH, Wetzlar, Germany) which is based on the Scheimpflug principle. In brief, using a camera a monochromatic slit-light source rotates together around the optical axes of the eye for measuring anterior segment tomography. Sectional images are collected and assembled to a 3-dimensional model. Scheimpflug imaging devices provide integrated staging and a series of corneal specific indexes that are commonly used in clinical settings [9]. Details of the Pentacam® HR are described elsewhere [11]. Only scans with a quality factor of > 95% were saved and further analyzed.

The following *topographic* Pentacam® HR anterior surface data were analyzed:

- Maximum simulated keratometry value in the central 3-mm-zone (Kmax)
- Inferior-superior value (I-S)
- Index of surface variance (ISV)
- Index of vertical asymmetry (IVA)
- Index of height asymmetry (IHA)
- Index of height decentration (IHD)

*Tomographic* Pentacam® HR parameters corneal thickness (CT), corneal astigmatism (CA) and mean 5–mm-zone and 7–mm-zone keratometry value (K5 and K7) (calculated from the total corneal refractive power map) were evaluated.

Furthermore, in all patients best-corrected distance visual acuity (CDVA), subjective spherical equivalent (SE), subjective sphere (Sph) and cylinder (Cyl) were recorded before and after surgery.

### Statistical analysis

Statistical analysis was performed using the software SPSS (version 23.0, Chicago, IL, USA). For comparing changes of variables before and 4 weeks after surgery, a mixed model was applied. Pearson correlation analysis was applied to check for possible associations between age, gender, corneal thickness and corneal biomechanical parameters. A *P* < 0.05 was considered as statistical significant.

## Results

The study included 42 eyes of 35 patients (20 female, 15 male). Mean age of all patients was 64.5 years (range 52–82 years).

**Fig. 1:**
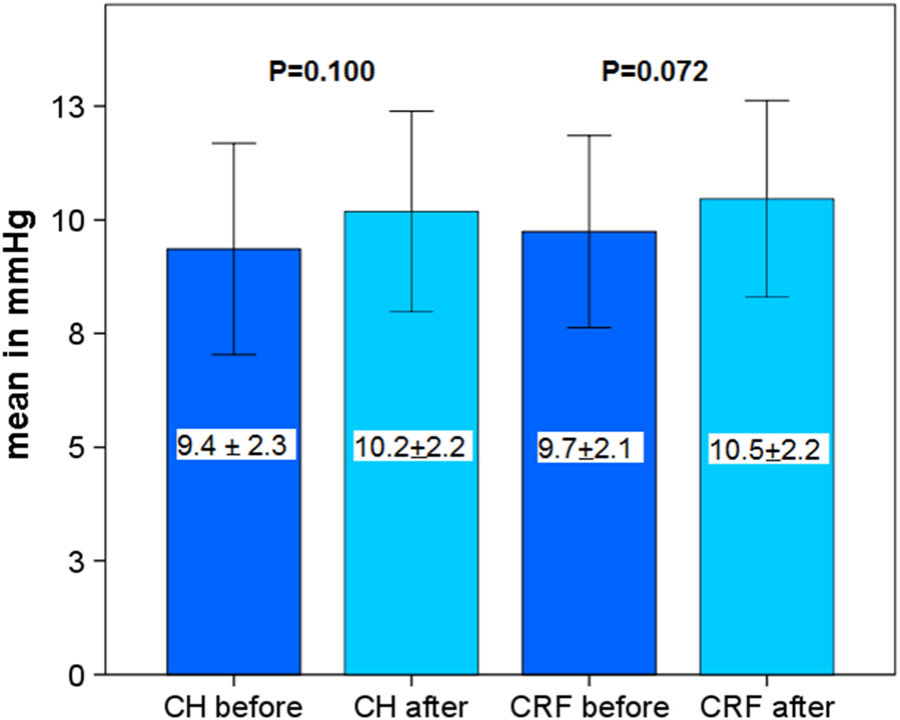
Corneal hysteresis (CH) and corneal resistance factor (CRF) before and 4 weeks after upper eyelid blepharoplasty

Four weeks after surgery CH and CRF increased (9.4 ± 2.3 to 10.2 ± 2.2 mmHg and 9.7 ± 2.1 to 10.5 ± 2.2 mmHg) but did not reach statistical significance (*P* = 0.100 and *P* = 0.072, Fig. 1).

**Fig. 2:**
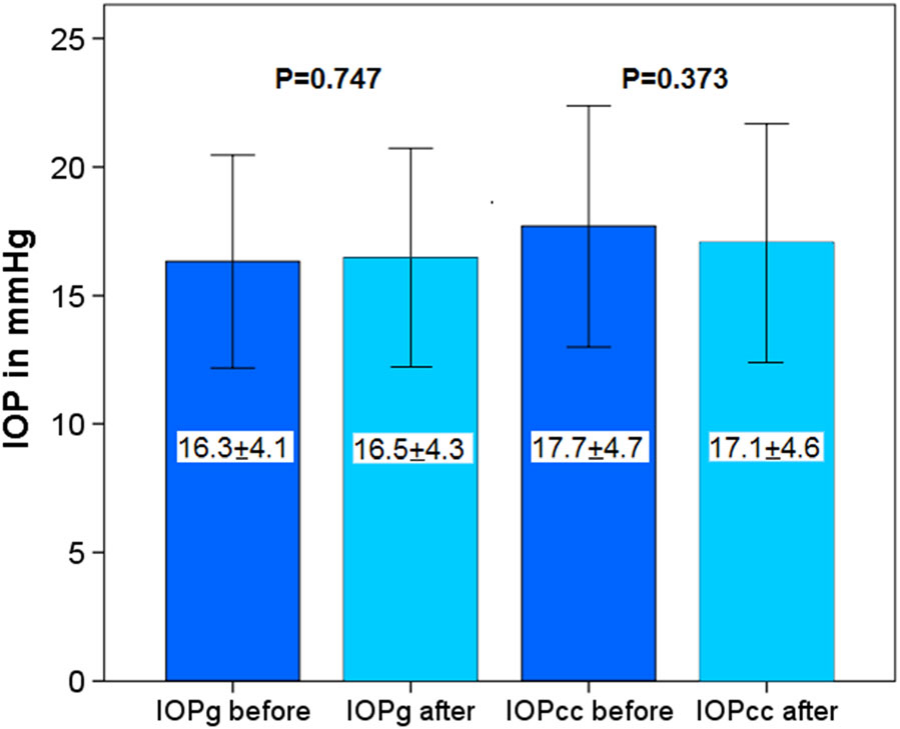
Goldmann-correlated IOP (IOPg) and corneal compensated IOP (IOPcc) before and 4 weeks after upper eyelid blepharoplasty

IOPg and IOPcc did not show statistically significant changes 4 weeks after blepharoplasty (*P* = 0.747 and *P* = 0.373, Fig. 2).

**Fig. 3:**
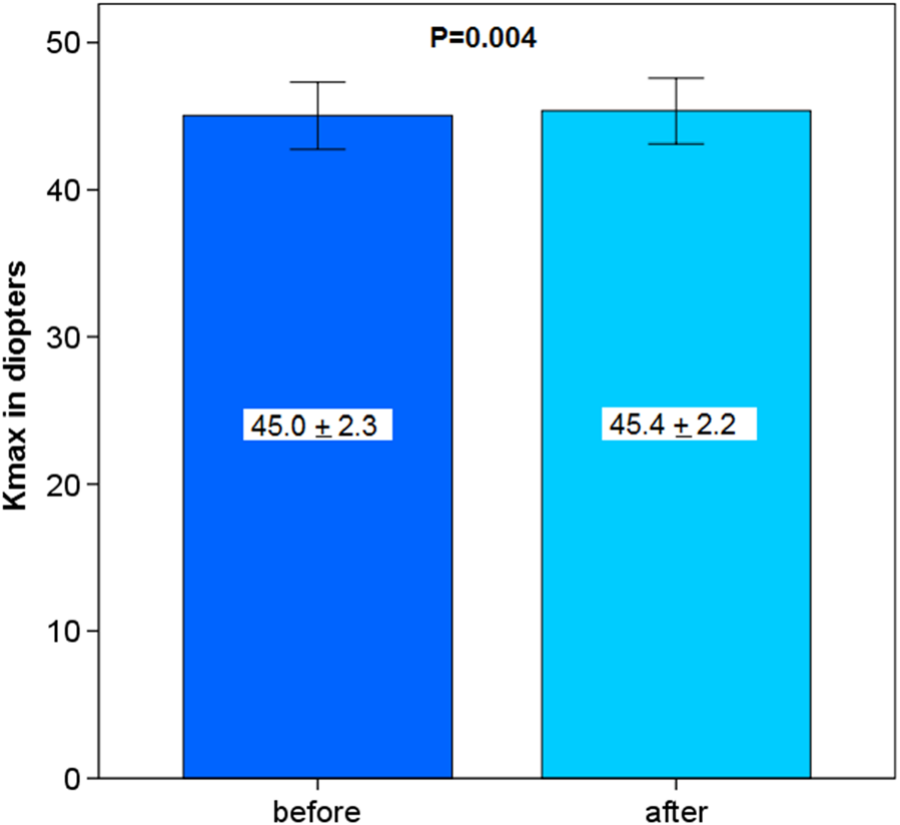
Maximum simulated keratometry value in the central 3-mm-zone (Kmax) before and 4 weeks after upper eyelid blepharoplasty

Kmax increased statistically significantly from 45.0 ± 2.3 to 45.4 ± 2.2 diopters (D) 4 weeks after blepharoplasty (*P* = 0.004, Fig. 3).

**Fig. 4:**
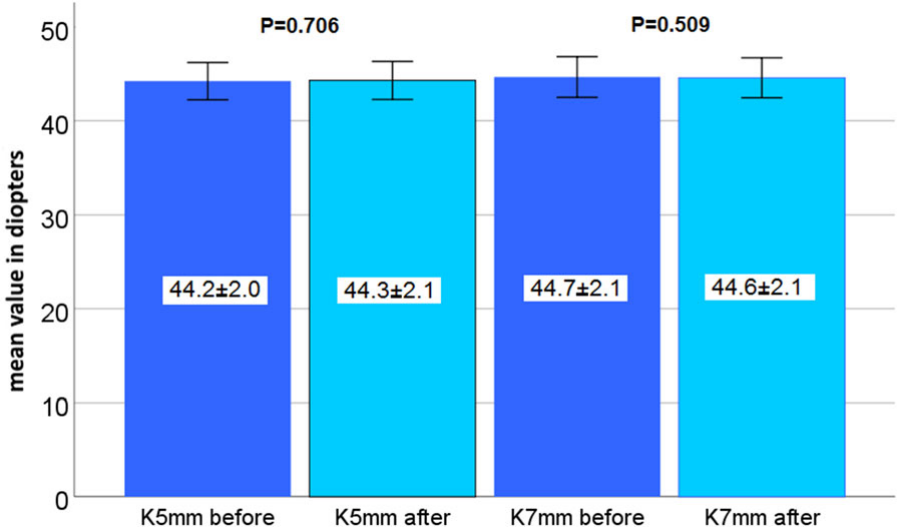
Mean 5-mm-zone keratometry (K5) and 7-mm-zone keratometry (K7) value before and 4 weeks after upper eyelid blepharoplasty

Mean keratometry indices for K5 and K7 showed no statistically significant change 4 weeks after surgery (44.2 ± 2.0 to 44.3 ± 2.1 D, *P* = 0.706 and 44.7 ± 2.1 to 44.6 ± 2.1 D, *P* = 0.509, Fig. 4).

**Fig. 5:**
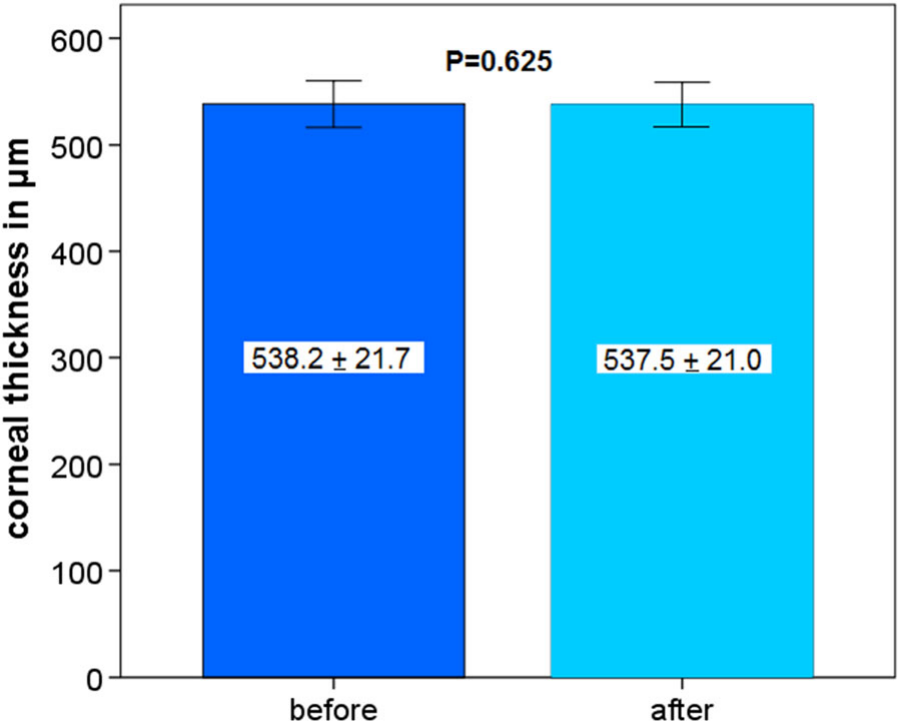
Corneal thickness before and 4 weeks after upper eyelid blepharoplasty

**Fig. 6:**
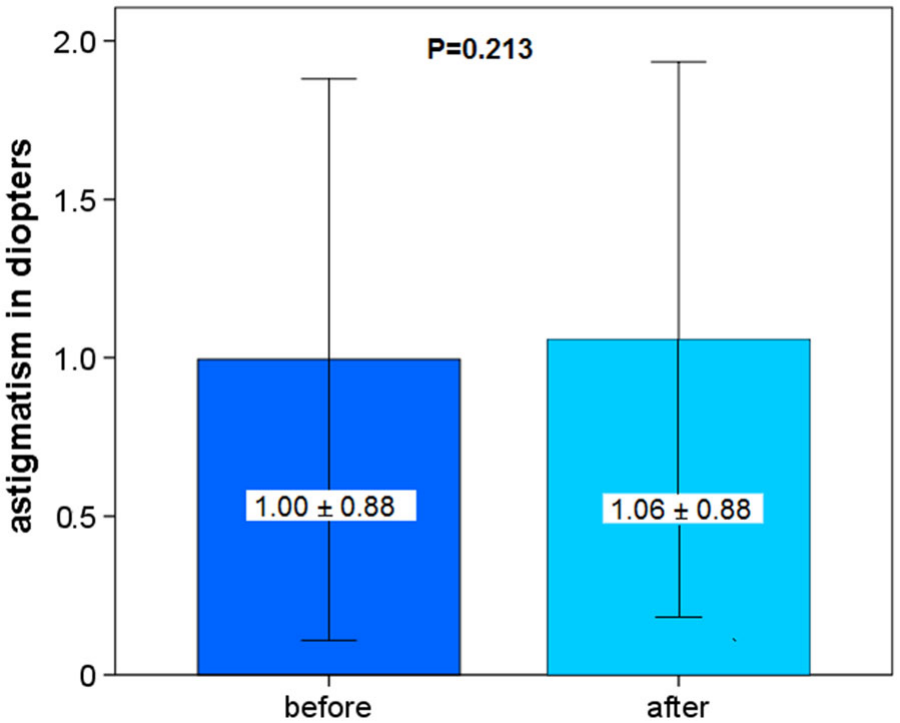
Corneal astigmatism before and 4 weeks after upper eyelid blepharoplasty

CT (at the thinnest point) and CA did not change significantly 4 weeks after blepharoplasty (*P* = 0.625 and *P* = 0.213, Fig. 5 and Fig. 6).

**Fig. 7:**
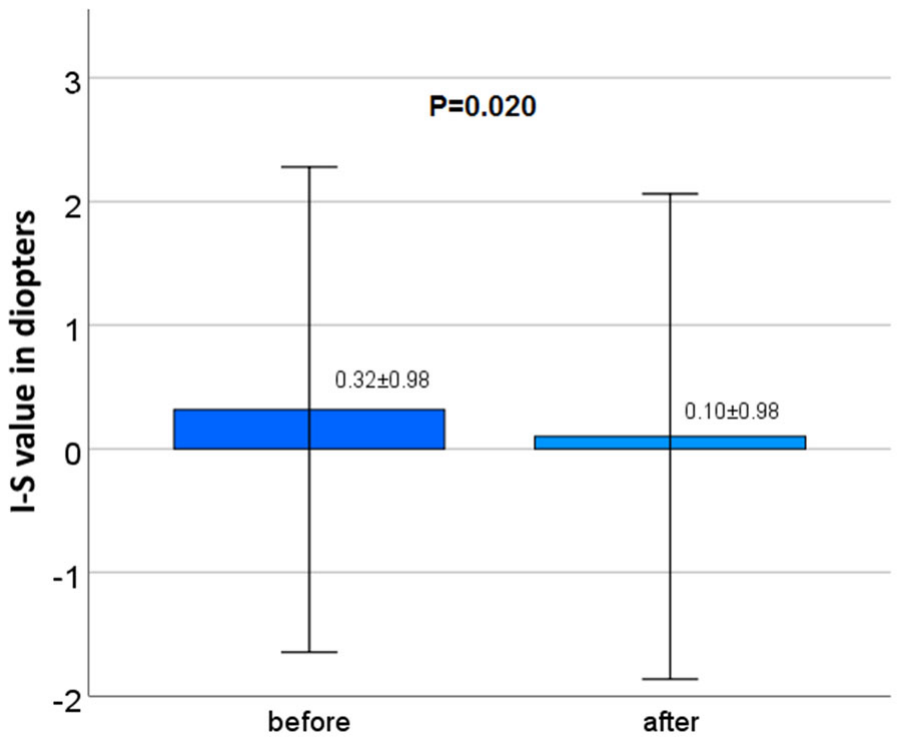
Inferior-superior value before and 4 weeks after upper eyelid blepharoplasty

The subjective cylindrical value changed significantly from -0.92 ± 0.91 to -1.04 ± 1.04 D, *P* = 0.038) and also I-S (0.32 ± 0.98 to 0.10 ± 0.98 D, *P* = 0.02) and ISV (19.98 ± 9.84 to 22.93 ± 11.23, *P* = 0.009) revealed significant changes after surgery (Table 1, Fig. 7).

**Table 1:** Changes in best-corrected distance visual acuity, subjective spherical equivalent, sphere, cylinder, inferior-superior value and Pentacam® HR corneal surface indices before and 4 weeks after upper eyelid blepharoplasty

Parameter	Before	After	P*
CDVA (logMAR)	0.11 ± 0.14	0.15 ± 0.15	0.142
SE (D)	− 0.09 ± 4.71	− 0.04 ± 4.51	0.437
Sph (D)	0.45 ± 4.50	0.44 ± 4.34	0.923
Cyl (D)	− 0.92 ± 0.91	− 1.04 ± 1.04	**0.038**
I-S (D)	0.32 ± 0.98	0.10 ± 0.98	**0.02**
ISV	19.98 ± 9.84	22.93 ± 11.23	**0.009**
IVA	0.16 ± 0.10	0.18 ± 0.09	0.088
IHA	7.27 ± 7.42	6.14 ± 6.24	0.120
IHD	0.01 ± 0.01	0.01 ± 0.01	0.307

CDVA, best-corrected distance visual acuity; SE, subjective spherical equivalent; Sph, subjective sphere; Cyl, subjective cylinder; I-S, inferior-superior value; ISV, index of surface variance; IVA, index of vertical asymmetry; IHA, index of height asymmetry; IHD, index of height decentration

logMAR, logarithm of the minimal angle of resolution; D, diopter

^*^Linear mixed model was used for continuous variables

^*^P < 0.05 represents statistical significance, significance is indicated in bold

No significant changes were detectable for the best-corrected distance visual acuity (0.11 logMAR ± 0.14 to 0.15 logMAR ± 0.15, *P* = 0.142), the subjective spherical equivalent (-0.09 ± 4.71 to -0.04 ± 4.51 D, *P* = 0.437), the subjective spherical refraction (0.45 ± 4.50 to 0.44 ± 4.34 D, *P* = 0.923) and the corneal surface indices IVA (0.16 ± 0.10 to 0.18 ± 0.09, *P* = 0.088), IHA (7.27 ± 7.42 to 6.14 ± 6.24, *P* = 0.120) and IHD (0.01 ± 0.01 to 0.01 ± 0.01, *P* = 0.307) (Table 1).

Age, gender and corneal thickness were not correlated with pre and postoperative differences of CH, CRF, corneal compensated IOP, Kmax, corneal astigmatism or I-S (Table 2).

**Table 2:** Correlation (Pearson) of pre- und postoperative differences with age, gender and preoperative corneal thickness (CT)

Parameter	Age	Gender	CT
Δ CH			
r	− 0.201	0.125	0.297
P	0.253	0.467	0.078
Δ CRF			
r	− 0.149	0.042	0.269
P	0.4	0.806	0.112
Δ IOPcc			
r	0.221	− 0.226	− 0.197
P	0.216	0.192	0.257
Δ Kmax			
r	0.025	0.112	− 0.018
P	0.887	0.509	0.916
Δ CA			
r	0.085	− 0.023	− 0.183
P	0.629	0.893	0.279
Δ I-S			
r	− 0.12	0.103	− 0.117
P	0.474	0.528	0.472

Δ, difference pre- to postoperative; CH, corneal hysteresis; CRF, corneal resistance factor; IOPcc, corneal compensated intraocular pressure; Kmax, central maximum simulated keratometry value; CA, corneal astigmatism; I-S, inferior-superior value

r: pearson correlation coefficient

*P* < 0.05 represents statistical significance

## Discussion

To detect a possible influence of topographic and tomographic parameters on the viscoelastic properties of the cornea after upper eyelid blepharoplasty, in the present study ORA measurements were performed before and 4 weeks after surgery. A previous study indicated a relationship between CH/CRF and corneal shape and corneal thickness [8]. In the present study, neither CH nor CRF showed a statistically significant change after blepharoplasty.

However, there was an increase of CH indicating a rise of corneal damping capacity. It might be hypothesized that the reduction of pressure on the cornea by a loss of eye lid weight may be responsible for the change in deformation of the cornea. The non-statistical increase of CRF could be important for goldmann applanation tonometry (GAT)—intraocular pressure measurements after upper eyelid blepharoplasty. A previous study reported a significant association between with CRF and GAT [12].

Reliable data on this topic are limited. To the best of (our) knowledge, the present study is the first to investigate the association of upper eyelid “skin-only” blepharoplasty and its effect on biomechanical properties of the cornea.

In the present study, it was shown that central Kmax was weakly but still statistically significantly affected by upper eyelid blepharoplasty 4 weeks after surgery with a mean change of 0.4 diopters in all patients. In contrast, the mean value of the total corneal refractive power of the 5 and the 7-mm-zone was not affected. Our results are in part consistent with findings of previously published data. A recently published study found similar results of 0.22 diopters difference between average preoperative and postoperative astigmatism one month after surgery [13]. Dogan et al. observed moderate but significant changes of corneal indices three months after blepharoplasty in patients with a margin reflex distance (MRD) of < 2.5 mm but not in those patients with a MRD > 2.5 mm [14]. All our patients showed a MRD of > 2.5 mm.

Brown et al. performed standard keratometry and corneal videokeratography measurements one and three months after blepharoplasty. In their follow-up, average dioptric changes of approximately 0.55 diopters with a broad variability were observed. The authors concluded that blepharoplasty may result in visually significant astigmatic changes in the central and peripheral cornea [6]. The fact of finding no changes of the peripheral total corneal power in the 5- and 7-mm-zone in our study is consistent with observations of Kim et al. They reported no significant changes in topography and astigmatism 4 weeks after surgery [15]. Also, Zinkernagel et al. described only mild changes of approximately 0.09 diopters in the “skin-only” blepharoplasty group but higher astigmatic changes in the blepharoplasty group with fat removal. The authors hypothesized that a larger fat reduction may also induce a more pronounced change of the corneal shape [16]. In the present study, we included only patients who underwent „skin-only” blepharoplasty. Removal of prolapsed upper lid fat compartments, resection of orbicularis oculi muscle or opening of the septum orbitale were exclusion criteria. Hence, we observed an increase in central but not in the paracentral corneal curvature.

We chose a follow-up of 4 weeks. After 4 weeks, the proliferative phase of wound healing is completed [17]. Using sutures to adapt dermal edges in direct apposition to epidermal and dermal layers, healing is primarily provided by epithelialization within the epidermis and with a limited extracellular matrix production in the dermis [18]. Hence, epithelialization and remodeling are negligible factors for a proper and tension-free adaption of the wounds after this time. A follow-up of 4 weeks was also chosen to detect a possible association of corneal changes and visual symptoms in the early postoperative period. Extending follow-up intervals may increase the risk of surgical-independent effects on corneal topography and viscoelastic changes. For these reasons, we did not schedule long term follow-ups.

Shao et al. observed persistent visual symptoms 1 year after upper eyelid blepharoplasty only in a small percentage and assumed that changes in the eyelid tension and pressure on the globe after surgery may be responsible for the symptoms [19].

In the present study, only one patient complained about temporary visual reduction, which, however, resolved completely three weeks after surgery.

CDVA did not show any statistically significant change after surgery which confirms that functional impairment only occurs in a small amount of patients. Subjective spherical equivalent and spherical refraction also remained unaffected which is consistent with findings of other studies [6, 7, 13].

Despite a statistically significant change in subjective cylindrical value, the mean increase of 0.12 diopters, however, does not seem to be clinically relevant.

I-S value describes differences of corneal power between the inferior and the superior hemisphere. A value of more than 1.4 diopters is suspicious for corneal ectasia [20].

In the present study, we were able to detect a statistically significant decrease of I-S which indicates the effect of blepharoplasty on the vertical corneal vector and a potentially stronger impact on the superior hemicornea compared to the inferior part. However, the I-S reduction of 0.22 diopters was very low. To the best of our knowledge, the I-S value has not been investigated in the context of blepharoplasty so far. Considering the statistically unchanged k values of the 5- and 7–mm-zone in our study, it may be concluded that upper eyelid blepharoplasty exerts a stronger effect on the elastic central cornea and the 3-mm-zone than on the paracentral and peripheral cornea.

Pentacam® HR software also proposes indices which characterize the irregularity of curvature of the anterior corneal surface (ISV), the degree of asymmetry between the curvature of the superior and the inferior cornea (IVA), the difference between the mean elevation of the superior cornea, the mean elevation of the inferior cornea (IHA) and the degree of vertical decentration of corneal elevation data (IHD) [20].

Apart from ISV, all other indices did not show any significant changes leading to the assumption that blepharoplasty has only a moderate effect on corneal curvature.

The statistically significant change of ISV does not seem to be clinically relevant since values were far below the pathologic level of > 41. However, there is an impact of blepharoplasty on the irregularity of the anterior curvature of the cornea.

Major limitation of the present study is certainly that dry eye symptoms were not objectively assessed. Despite exclusion of patients who complained about dry eye symptoms and applied artificial eye drops, an objective investigation with specific measurement tools was not performed. Hence, the impact of this important factor on measurement results remains unclear. Secondly, it can be assumed that an increase of number of participants might have led to statistically significant results, especially for the parameters CH and CRF.

## Conclusions

In conclusion, the trend of increasing CH and CRF values might indicate a rise of corneal damping capacity. It might be hypothesized that the reduction of pressure on the cornea by a loss of eye lid weight may be responsible for the change in deformation of the cornea. Statistically significant alterations of Kmax, I-S and ISV were observed. The corneal steepening with a mean change of 0.4 diopters and the decrease of I-S with a mean of 0.22 diopters do not have a clinically relevant effect for “skin-only” blepharoplasty patients in daily practice.

## Data Availability

The data will be available in the case of reasonable request by the corresponding author.
